# A Rare Site of Metachronous Metastases from Renal Cell Carcinoma

**DOI:** 10.15586/jkcvhl.v11i1.282

**Published:** 2024-01-05

**Authors:** Madhuri Nutakki, Kanchan. V. Murhekar, Shirley Sundersingh, Anand Raja

**Affiliations:** Department of Surgical Oncology, Cancer Institute (WIA), Chennai, India

**Keywords:** renal cell carcinoma, testis, metastasis, nephrectomy, orchidectomy

## Abstract

Secondary metastatic involvement of the testis is a rare occurrence, particularly in cases of metastasis from renal cell carcinoma (RCC). We present a case of metachronous contralateral testicular metastasis from RCC in a 55-year-old man, occurring 2 years after radical nephrectomy. Following a thorough evaluation that ruled out systemic disease, the patient underwent a Chevassu procedure and right inguinal orchidectomy. Histopathological analysis confirmed metastatic involvement of the right testis by RCC. Metastasis to the testis from RCC is uncommon, with only a few cases reported in the literature. Isolated metachronous metastasis without systemic involvement is even rarer. This case highlights the importance of considering testicular metastasis in patients with a history of RCC, emphasizing the need for comprehensive evaluation and surgical resection when feasible, as it has been associated with prolonged survival.

## Introduction

Metastatic carcinoma affecting the testis is a rare occurrence, with prostatic carcinoma being the most common primary tumor to metastasize to the testis. Other primary sites include the lung, malignant melanoma, colon, and kidney (1). Isolated metastasis to the testis from primary renal cell carcinoma (RCC), without systemic involvement, is exceptionally rare (2).

## Case Report

A 55-year-old gentleman had been diagnosed with a nonmetastatic left renal tumor, for which he underwent an open left radical nephrectomy. The postoperative histopathology was suggestive of a clear cell variant of RCC invading the renal vein, pT3N0M0. Postoperatively the patient was on regular follow-up. After a disease-free survival (DFS) of 24 months, the patient presented with a history of progressive painless enlargement of the right hemi-scrotum. There was no history of any trauma or fever.

**Figure 1:**
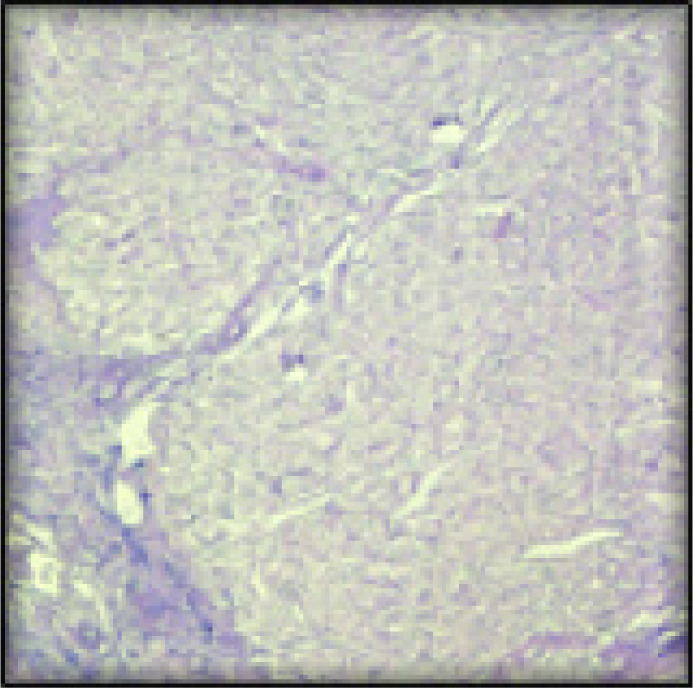
Renal cell carcinoma: Tumor comprising round cells with abundant clear cytoplasm and central pyknotic nucleus. Clear cell variant, NG low.

**Figure 2:**
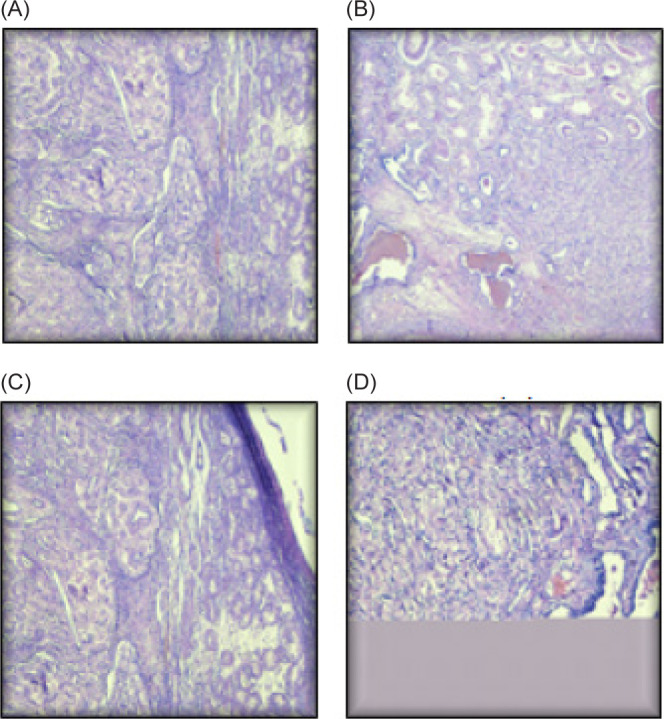
Testis infiltrated by RCC (A,B,C,D) – testicular parenchyma with seminiferous tubules is seen on the right side of the field. Left side shows the tumor.

On examination, the patient had performance score of ECOG 0. Local examination revealed a nontender multi-nodular lesion in the right scrotum with normal left scrotum, firm in consistency, with no fluctuation, transillumination, or cough impulse. The skin over the scrotum was normal and free. Physical examination was otherwise unremarkable. An ultrasound of the testis revealed a normal left testis with multiple hypoechoic lesions in the right testis, the largest measuring about 3×2 cm with increased vascularity on Doppler with normal epididymis. Serum tumor markers for germ cell tumors (beta-HCG, AFP, LDH) were within normal limits. Contrast-enhanced computerized tomography (CECT) of the abdomen and pelvis did not show any abnormality in the abdomen or pelvis, except for the tight testicular lesion. The chest radiograph was normal. Positron emission tomography (PET-CT) did not show any area of abnormal metabolic uptake except the right testis. The patient underwent a Chevassu maneuver, and intraoperative frozen was suggestive of metastatic deposits in testicular parenchyma, probably from renal carcinoma. The patient underwent a high inguinal orchidectomy in the same sitting. The final histopathology showed multiple nodules in the testis, the largest measuring about 2×2×1.5 cm, involving the capsule of the testis, with normal testicular parenchyma identifiable in between. The lesion was suggestive of metastatic deposits from RCC – clear cell variant (Figures 1 and 2), which was consistent with the primary in the left kidney. Given the solitary metastasis and following the complete resection of the same, the case was discussed in the multidepartment tumor board. It was decided to keep the patient under close follow-up. The patient is doing well with no evidence of recurrence elsewhere at a follow-up of 36 months.

## Discussion

While RCC frequently metastasizes to various organs, testicular spread is exceedingly rare. The testes are considered a “tumor sanctuary,” making the establishment of metastatic tumor cells unlikely due to the scrotum’s relatively low temperature (3). For most patients, RCC remains an organ-confined disease, and surgical resection results in excellent survival outcomes (4, 5). However, there are still treatment challenges in those with advanced or metastatic stage of the disease (6). Approximately 25–30% of patients present with metastatic disease, while 20–40% of men and women who undergo surgical resection for localized RCC will develop metastases (7, 8). The incidence of metastatic deposits in the testis from renal carcinoma is very low (0.06 to 1.6%) (9). The first case of RCC metastasis to the testis was described by Bandler et al. in 1946 (10). Saitoh et al. in their autopsy study of 1451 RCC patients found metastatic disease in 89% of whom 81% had multiple sites of metastatic involvement, but none had involvement of testis (11).

Testicular metastases can present synchronously or metachronously with renal carcinoma. Testicular swelling can rarely be the presenting feature of RCC with diagnosis being made after orchidectomy (12, 13). Cases of metachronous involvement of the testis after a long disease-free interval post-nephrectomy have also been described (14, 15). Wang et al. described five case reports among which, one patient had testicular mass as the initial presentation leading to a diagnosis of RCC, three had ipsilateral metastases, one had contralateral metastasis, and one had bilateral metastasis (16).

Clear cell variant of RCC is the commonest histological type to metastasize to the testis; rarely the chromophobe variant may also metastasize (14, 17). Metastases to either side can occur, however. Ipsilateral metastasis is more commonly described, with the left side being affected more often. The exact etiology of the mode of spread to the testis is not clearly known, especially in cases of isolated testicular metastasis. Retrograde extension along the gonadal veins into the testis is one plausible explanation. It is more consistent for left-sided testicular metastasis. Other modes of spread like spread via vertebral plexus of Baxton, arterial embolization may also play a role, especially in contralateral metastasis (15).

If a solitary recurrence is detected, the best treatment is surgical excision, regardless of whether it is synchronous or metachronous (17). Surgical excision is the recommended treatment for solitary testicular metastases, as it has been associated with improved long-term survival (18).

## Conclusion

Metastatic involvement of the testis from primary RCC is a rare occurrence with unique diagnostic and therapeutic test of. A high index of suspicion is necessary to identify secondary deposits, and a comprehensive evaluation is crucial due to the potential for multicentric spread. Surgical resection should be considered in cases of solitary metastasis, as it has been found to result in prolonged survival.
